# Microwave-Assisted Synthesis of Pd Nanoparticles into Wood Block (Pd@wood) as Efficient Catalyst for 4-Nitrophenol and Cr(VI) Reduction

**DOI:** 10.3390/nano13172491

**Published:** 2023-09-04

**Authors:** Zhao Zhang, Arnaud Besserer, Christophe Rose, Nicolas Brosse, Vincent Terrasson, Erwann Guénin

**Affiliations:** 1 Université de Technologie de Compiègne, ESCOM, TIMR (Integrated Transformations of Renewable Matter), Centre de Recherche Royallieu, CS 60319, 60203 Compiègne CEDEX, France; zhao.zhang@utc.fr; 2 LERMAB, Université de Lorraine, INRAE, F54000 Nancy, France; arnaud.besserer@univ-lorraine.fr (A.B.); nicolas.brosse@univ-lorraine.fr (N.B.); 3 Centre INRAE-Grand Est-Nancy, UMR SYLVA-SILVATECH pole IM3, 54280 Champenoux, France; christophe.rose@inrae.fr

**Keywords:** nanocatalysis, wood catalyst support, microwave wood pretreatment, wastewater treatment, reusability

## Abstract

Palladium (Pd) nanoparticle catalysis has attracted increasing attention due to its efficient catalytic activity and its wide application in environmental protection and chemical synthesis. In this work, Pd nanoparticles (about 71 nm) were synthesized in aqueous solution by microwave-assisted thermal synthesis and immobilized in beech wood blocks as Pd@wood catalysts. The wood blocks were first hydrothermally treated with 10% NaOH solution to improve the internal structure and increase their porosity, thereby providing favorable attachment sites for the formed Pd nanoparticles. The stable deposition of Pd nanoparticle clusters on the internal channels of the wood blocks can be clearly observed. In addition, the catalytic performance of the prepared Pd@wood was investigated through two model reactions: the reduction of 4-nitrophenol and Cr(VI). The Pd@wood catalyst showed 95.4 g^−1^ s^−1^ M^−1^ of normalized rate constant k_norm_ and 2.03 min^−1^ of the TOF, respectively. Furthermore, Pd nanoparticles are integrated into the internal structure of wood blocks by microwave-assisted thermal synthesis, which is an effective method for wood functionalization. It benefits metal nanoparticle catalysis in the synthesis of fine chemicals as well as in industrial wastewater treatment.

## 1. Introduction

Nanocatalysts, and especially metal nanoparticles catalysis, are increasingly used [1,2]. They offer many advantages over traditional catalysts due to their unique properties, improved volume-to-surface ratio and better catalytic performance [3,4]. For example, noble metal nanoparticle catalysts are used for environmental remediation to remove toxic organic pollutants such as nitro-aromatic compounds, dyes or toxic metals used in industry from wastewater [5,6,7]. In particular, Pd nanoparticles have been widely used in coupling reactions, catalytic hydrogenation and oxidation reactions, as well as hydrogen storage materials and catalytic electrodes for fuel cells in recent years [8,9,10].

However, despite the high efficiency of Pd nanoparticle catalysts, their use is often hindered by their lack of stability in the reaction medium and their low recyclability. To circumvent this problem, the immobilization of nano-catalysts on supports is a promising solution. Evans et al. [11] investigated the effects of the metal-oxide supports (CeO_2_, SnO_2_, TiO_2_) in the catalytic activity of Pd nanoparticles for ethanol electrooxidation. Mahmoud et al. [12] summarized the progress on Pd nanocatalysts supported on different polymers (MOFs polymer, PVP/alumina, functional resins, etc.) for selective and sustainable oxidation of alcohols, olefins, alkenes, etc. Moreover, other supports can be obtained from renewable materials or wastes, which introduces more sustainability in the process [13,14,15]. Dong et al. [16] successfully prepared Pd catalysts based on carbene functionalized cellulose as a carrier for the Suzuki reaction in aqueous solution. Kumar et al. [17] used lignin to synthesize and immobilize Pd nanospheres in water without any reducing agent for Heck reaction under solvent-free conditions. Marjan et al. [18] developed a green approach for the ultrasound promoted in situ immobilization of Pd NPs over chitosan/agarose modified Fe_3_O_4_ as an effective catalyst for the oxidation of alcohols.

In recent years, many approaches have been developed for the functionalization of natural wood, due to the fact that it is the most abundant renewable biomass in nature [19,20]. However, both the extraction of cellulose and lignin from wood and their chemical modification still pose a great challenge for the highly efficient utilization of wood [21,22]. Furthermore, it is still difficult to achieve the same level of complexity in the internal structure of natural wood using synthetic approaches. Therefore, functionalization of natural wood is a promising alternative, as it directly leverages its inherited architecture [23,24]. It effectively combines the intrinsic properties of natural wood with new specialized functionalities. Furthermore, the main components of the wood cell wall contain lignin, cellulose and hemicellulose [25,26]. The large number of phenolic hydroxyl and ether groups in lignin and cellulose are very favorable for the formation of Pd nanoparticles [27]. Moreover, the complex internal channels and numerous pores of wood can provide attachment sites for Pd nanoparticles. In addition, the reusability of catalysts is of great importance in chemistry and industry [9,12]. The reusability of natural wood blocks is a significant advantage when using them as supports for Pd nanoparticles. A simple filtration operation enables the wood blocks to be easily recovered without complex experimental techniques. This feature makes the recycling and reuse of catalysts more cost-effective and helps to reduce the environmental impact.

In this study, Pd nanoparticles were synthesized and immobilized in natural wood blocks as Pd@wood catalyst by microwave-assisted thermal synthetic methods in aqueous solution. The internal microstructures of the wood blocks without treatment and after pretreatment with NaOH solution were characterized by Scanning Electron Microscopy (SEM). The size distribution of Pd nanoparticles deposited inside the wood block was characterized by SEM and EDS. In addition, model reaction tests were then performed to evaluate the catalytic performance of the prepared catalysts, such as the reduction of 4-nitrophenol and Cr(VI).

## 2. Materials and Methods

### 2.1. Materials

Sodium tetrachloropalladate trihydrate (Na_2_PdCl_4_), ethanol (96% vol), acetone, potassium dichromate (K_2_Cr_2_O_7_), formic acid, sodium hydroxide (NaOH), HNO_3_ (67–69%, super pure for trace analysis), HCl (37%, trace metal analysis), palladium standard solution (for AAS, 1 mg/mL Pd in 10% HCl), sodium borohydride (NaBH_4_), benzyl phosphonic acid, 4-nitrophenol and ascorbate sodium were purchased from Thermo Fisher Scientific in France and used as received. Wood blocks (beech) was purchased from a hardware store in France. Deionized water generated from the purification chain was used for all experiments.

### 2.2. Characterization Methods

The internal structure of the wood blocks and the distribution of Pd nanoparticles inside the wood block were characterized by Scanning Electron Microscopy (FEG-SEM Sigma HD VP model from Zeiss Company, Jena, Germany). X-ray micro-analysis (EDS, Energy Dispersive Spectrometry) was used to confirm the presence and chemical composition of Pd NPs. Wood samples were previously coated with very low thickness of Carbon sputtering (ACE 600 Leica, Wetzlar, Germany). The observations and characterization of Pd nanoparticles were realized by SEM with high resolution in lens detector in High Vacuum mode, low kV of acceleration beam (5 kV) and at high magnification. EDS spectroscopy (SDD Xmax 80 mm² spectrometer, Oxford Instruments, Manchester, UK) was used to characterize the chemical composition (elementary Pd) of nanoparticles at the scale of the SEM magnification. The internal structure and morphology of wood blocks before and after NaOH pretreatment were characterized by SEM (FEI Quanta FEG 250, Hillsboro, OR, USA) with accelerating voltage of 20 kV. The UV-Visible spectrum was performed on a spectrometer Lambda 12 (PERKIN ELMER, Waltham, MA, USA) instrument in the wavelength range of 250–800 nm with a scanning speed of 480 nm/min and a bandwidth of 5 nm. The loading of Pd nanoparticles (mol%) was measured by the instrument ICP (Agilent 4200 MP-AES, Santa Clara, CA, USA). The details are as follows: firstly, a series of Pd standard solutions of different concentrations (50, 100, 125, 200, 250 ppm) was prepared to calibrate the ICP equipment and obtain the standard concentration curve. Volumes of 0.5 mL of Pd standard solution, 1.0 mL of HNO_3_, 2.0 mL of HCl and 6.5 mL of deionized water were added to a 10 mL standard volumetric flask to prepare a Pd calibration solution with a concentration of 50 ppm. Volumes of 1.0 mL of Pd standard solution, 1.0 mL of HNO_3_, 2.0 mL of HCl and 6.0 mL of deionized water were used to prepare a Pd calibration solution with a concentration of 100 ppm. Other concentrations of Pd calibration solutions were prepared as indicated above. The amount of Pd supported in the wood block with a length of 1.0 cm was calculated by subtracting the remaining Pd content in the solution after wood block loading from the total content of Pd in the initial solution. Volumes of 2.0 mL of the reacted solution, 1.0 mL of HNO_3_, 2.0 mL of HCl and 5.0 mL of deionized water were added to the beaker and then used for further ICP analysis, which was to determine the concentration of Pd remaining in the solution.

### 2.3. Pretreatment of Wood Blocks

Wood blocks from beech of three different lengths (0.5, 1.0, 1.5 cm) with a diameter of 1.0 cm were prepared by hand sawing. These blocks were then treated in sodium hydroxide solution (10%) at 80 °C for 2 days. After that, they were thoroughly washed three times with distilled water and dried in an oven.

### 2.4. Preparation of Pd@wood Catalyst

The Pd nanoparticles were prepared by using a protocol involving the reduction of Pd salts by sodium ascorbate in the presence of a benzyl phosphonic acid as stabilizer, following a process adapted from A. Iben Ayad et al. [28]. Briefly, benzyl phosphonic acid solution (0.07 mol/L) was prepared in distilled water, before adding NaOH (1 M) to adjust the pH of solution to 10. Pd salt solution (0.0038 mol/L) was prepared by dissolving Na_2_PdCl_4_ in distilled water. Sodium ascorbate solution (0.087 mol/L) was prepared in distilled water. Then, 10 mL of distilled water, 1.5 mL of Pd salt solution, 900 µL of stabilizer solution, 200 µL of sodium ascorbate solution and a 1.0 cm long piece of wood were added sequentially to a 30 mL reaction tube. The tube was then placed in a Microwave Synthesis Reactor (Monowave 300, Anton Paar France SAS, Les Ulis, France) and heated in 1 min to 100 °C and kept at this temperature for 30 min with a stirrer speed of 1200 rpm. A rapid heating program in the Microwave Synthesis Reactor was used (Figure S1). For the first 12 s, the maximum power reached 678 W, and then it started to decrease. Within 1 min, the temperature reached 100 °C, and the power was maintained between 20 W and 30 W during the rest of the process. After the reaction was completed, the tube was removed from the microwave reactor and then cooled to room temperature. Finally, the blocks were removed and dried in an oven before being used for further analysis.

### 2.5. General Procedure for Reduction of 4-Nitrophenol

The 4-nitrophenol aqueous solution of 5 mL concentration of 0.807 mmol/L was first prepared in a beaker. Then the freshly prepared NaBH_4_ solution of 1 mL concentration of 1.69 mol/L was added to the beaker and stirred at the speed of 800 rpm. After 5 min, 200 μL of the solution was taken out for UV analysis as the initial concentration of 4-nitrophenol. Afterwards, the wood block of 1.0 cm was added to the beaker. At a certain time, 200 μL solution was taken out for UV analysis. After each reaction cycle, the Pd@wood composite sample was washed with water and acetone, then treated by drying or sonication before the next cycle use.

### 2.6. General Procedure for Reduction of Cr (VI)

The reduction of Cr (VI) was carried out following the report of Dandapat et al. [29] and modified as appropriate. Briefly, K_2_Cr_2_O_7_ aqueous solution of 5 mL concentration of 5 mmol/L was first prepared in a beaker. Then 1 mL of formic acid was added to the beaker and the temperature was kept at 50 °C. After 3 min, 0.5 mL of the solution was taken out for UV analysis as the initial concentration of Cr(VI). Afterwards, the wood block of 1.0 cm was added to the beaker. At a certain time, the 0.5 mL reaction solution was taken out for UV analysis. After each reaction cycle, the Pd@wood composite sample was ultrasonically washed with water and acetone, then dried in an oven at 40 °C before the next cycle use.

## 3. Results and Discussion

### 3.1. Structural Characterization of Catalyst

Natural wood blocks are used as supports for Pd nanoparticles to form composite catalysts. The use of wood blocks as support has the potential following advantages: (1) Wood is a renewable and environmentally friendly support, which does not require complex preparation. (2) Wood possesses a complex porous structure with an abundance of pores and surface area. This structure contributes to the good dispersion of Pd nanoparticles, providing more active sites and thus increasing the activity of the catalyst. Moreover, it allows for good adsorption of the pollutants that can diffuse in the structure. (3) The complex internal structure of the wood blocks can protect the Pd nanoparticles. preventing them from falling off during the catalytic process. (4) Retrievability of the catalyst is also greatly simplified.

**Figure 1:**
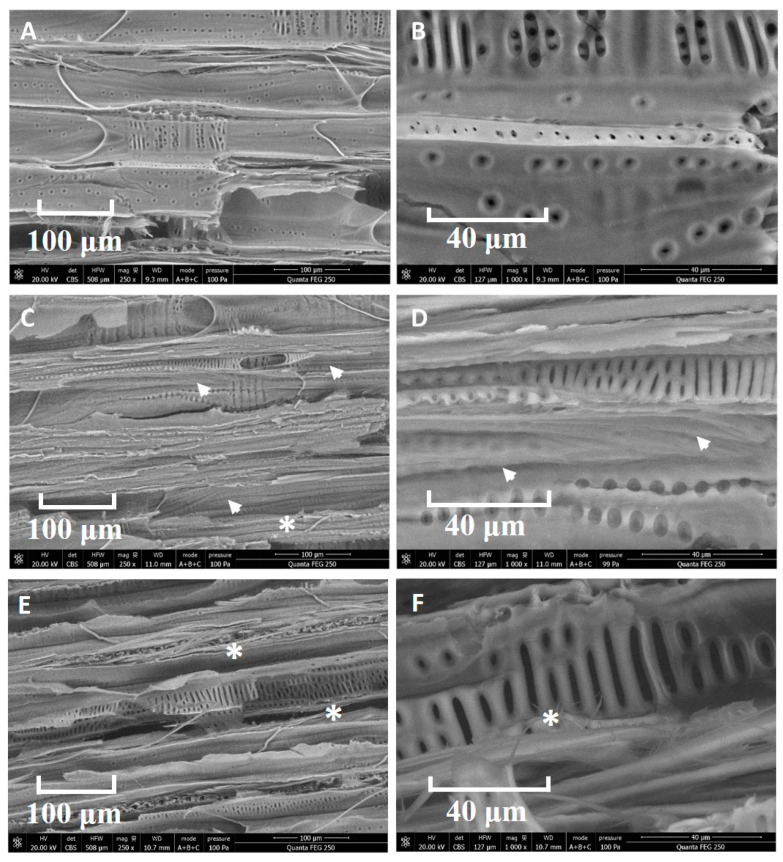
SEM images of the internal structure of wood blocks. (**A**,**B**) Untreated samples. (**C**,**D**) After NaOH treatment. (**E**,**F**) After NaOH and Pd nanoparticles impregnation. Notes the cell wall alterations (arrowheads) and defibration (asterisks).

The effect of the NaOH pretreatment and the Pd nanoparticles impregnation on the internal microstructure of the wood blocks was investigated by SEM. Representative images of specimens are shown in Figure 1. Compared with untreated samples, numerous grooves are visible on vessel cell walls in NaOH-treated samples. Slight defibration was also noticed. It was shown that alkaline hydrothermal pretreatment of wood triggered the removal of lignin from the cell wall [30]. This is because, in general, the ester bonds between lignin, hemicellulose and cellulose may be broken after alkali treatment of biomass [31]. Hydrothermal treatment significantly changed the total porosity of yellow poplar (Liriodendron tulipifera Linnaeus) samples [32] and the porosity of biomass was increased after NaOH thermal pretreatment of wood blocks [33]. These intricate channels and pores in wood cell wall might provide perfect attachment points for Pd nanoparticles. Alterations of middle lamella, lignin and porosity depend on the density and anatomical features of the wood species [34]. Beech (Fagus sylvatica) wood is a diffuse porous wood with a high potential for impermeability according to the European Standard EN 350 [35].

**Figure 2:**
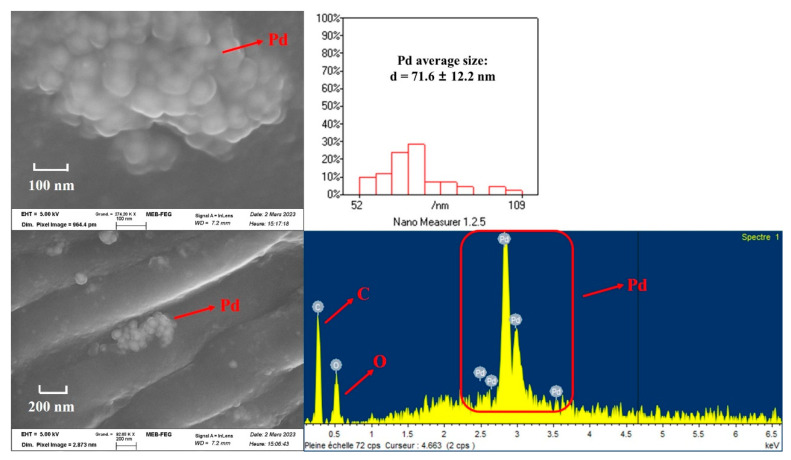
SEM images and EDS spectrum of the internal structure of Pd@wood catalyst, and the size distribution of Pd nanoparticles.

The SEM images and EDS spectrum of the internal structure of the wood block after deposition of Pd nanoparticles are presented in Figure 2. It can be observed that the Pd nanoparticles clusters are distributed on the internal channels of the wood block. Two strong signal peaks of Pd element appear on the EDS spectrum, which indicates the in-situ synthesis of Pd nanoparticles inside the wood block. It turned out that the pretreated wood blocks provided favorable sites for attachment of Pd nanoparticles. Furthermore, the average size is about 71 nm by the size distribution of Pd nanoparticles, which indicates that the formation of Pd clusters due to agglomeration of Pd nanoparticles. The loading of Pd nanoparticles in the 1.0 cm wood block was measured and calculated. The remaining Pd concentration in the solution after wood block loading was 8.76 ppm by ICP analysis. The mass of residual Pd in solution and the initial total mass of Pd were calculated to be 0.552 mg and 0.716 mg, respectively. Thus, the loading mass of Pd in the 1.0 cm wood block was 0.164 mg (1.54 × 10^−3^ mmol).

### 3.2. Study of Catalytic Properties

**Figure 3:**
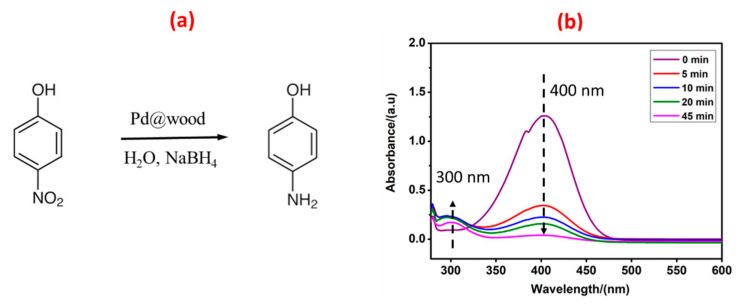
(**a**) The reduction of 4-nitrophenol to produce 4-aminophenol. (**b**) UV–vis spectra for reduction of 4-nitrophenol and formation of 4-aminophenol, treated with 1.0 cm wood catalyst.

The new catalyst supported on wood blocks was evaluated in two model reactions for depollution: reduction of 4-nitrophenol and Cr(VI). These two model reactions are important for industrial water purification and environmental protection due to their toxicity to humans and nature [36]. The catalytic activity of the prepared Pd@wood catalyst was first investigated by the reduction reaction of 4-nitrophenol, as shown in Figure 3. The reduction of 4-nitrophenol is a well-known process, in which 4-nitrophenol is reduced to 4-aminophenol [37] (Figure 3a). In our study, NaBH_4_ was the reducing agent and Pd nanoparticles were used as the catalyst. The pH of the catalytic media for the reduction process of 4-nitrophenol is 10.7. UV spectroscopy was used to record the reaction process (Figure 3b). The peak at 400 nm is attributed to the absorption of 4-nitrophenolate ion, while the peak at 300 nm is due to the absorption of 4-aminophenol. As the reaction time increases, the peak intensity at 400 nm decreases while that at 300 nm increases. This demonstrates the reduction of 4-nitrophenol and the formation of 4-aminophenol. This is consistent with the results reported in the literature [38,39]. The reduction was moreover confirmed by NMR of the isolated product (Figure S5). It can be observed that 1.0 cm wood catalyst could completely reduce 4-nitrophenol after 45 min. It is attributed to the fact that the reaction substrate can easily enter the internal channels of the wood block to contact with Pd nanoparticles and thus be reduced. However, the decrease in 4-nitrophenol could not be ascribed to only adsorption of the molecule onto wood as it is less rapid than the reduction process (see Figure S4a,b).

**Figure 4:**
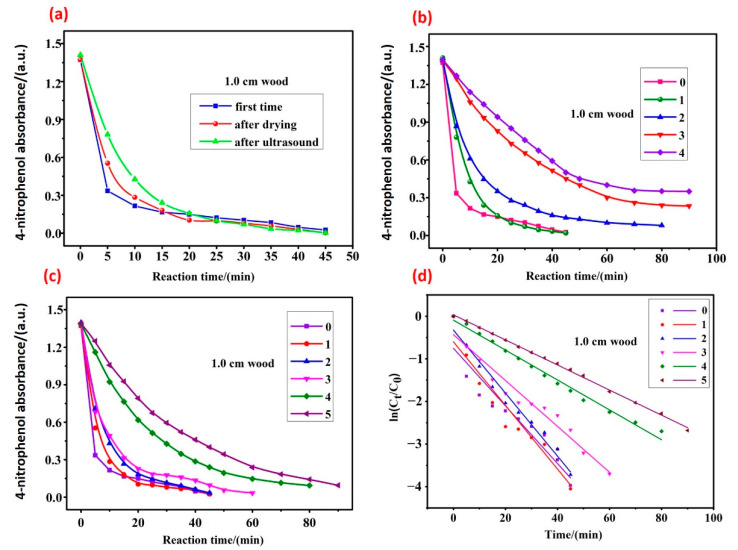
Catalytic reduction of 4-nitrophenol. (**a**) Treated with 1.0 cm wood catalyst after drying and sonication, respectively. (**b**) Treated with 1.0 cm wood catalyst only by ultrasonic cleaning after each reaction for four cycles. (**c**) Treated with 1.0 cm wood catalyst by ultrasonic cleaning followed by drying after each reaction for five cycles. (**d**) Kinetic curves of (**c**), corresponding plots of ln(C_t_/C_0_) versus reaction time.

At the end of each reaction, the blocks were removed and washed three times with distilled water and acetone, and then the blocks were treated by drying and ultrasonication before the next catalytic reaction. It was observed that after the first drying or sonication, the wood block was still able to reduce the 4-nitrophenol completely within 45 min (Figure 4a). As the number of cycles increased, the time required for the reduction reaction gradually increased. In the case of only ultrasonic cleaning after each reaction (Figure 4b), the first cycle could still reduce 4-nitrophenol by 100%, but the second cycle could reduce 4-nitrophenol by 94.3%. In the following two cycles, only 83.2% and 74.9% of the reactant were reduced, respectively. However, in the case of ultrasonic cleaning followed by drying after each reaction (Figure 4c), 4-nitrophenol could be completely reduced in the first three cycles. Under the fourth and fifth cycles, the reduction rate of 4-nitrophenol remained as high as 93.3% and 93.1%, respectively. The main reason was that the obstruction of the pores of the wood by the reaction products, which explained that drying and ultrasonic cleaning had a positive effect in recycling.

In order to study the catalytic efficiency of the prepared Pd@wood catalyst for reducing 4-nitrophenol, the rate constant k_app_ (s^−1^) is calculated based on the equation: k_app_ = −(d(ln(C_t_/C_0_)))/dt, where t (s) is the reaction time, and C_t_ and C_0_ represent the concentration of 4-nitrophenol at the reaction time t and the initial time t_0_, respectively [40]. The calculated reaction rate constant k_app_ was 4.41 × 10^−3^ s^−1^ for the wood composite catalyst with a length of 1.0 cm and Pd loading of 0.164 mg. For the purpose of comparing with the reported catalyst, the constant normalized k_norm_ (s^−1^) including the concentration of NaBH_4_ and the quantity of Pd should be considered; it is calculated based on the equation: k_norm_ = k_app_/(m(Pd) × (NaBH_4_)), where m(Pd) refers to the quantity of Pd and c(NaBH_4_) represents the concentration of NaBH_4_ [41]. The calculated normalized rate constant k_norm_ was 95.4 g^−1^ s^−1^ M^−1^. Comparison of different catalyst systems on the reduction of 4-nitrophenol is shown in Table 1. It can be observed that higher normalized rate constant value and less catalyst was used in our case, compared to other catalytic systems Ag@lignin [40], Cu NPs [38] and Ag dendrites [42]. In addition, the prepared Pd@wood catalyst exhibited higher or comparable values of k_app_ and k_norm_ than Pt black and Au@citrate [43]. The exception is the Pd nanodendrite (PdND1) prepared by Mourdikoudis et al. [41], but it should be noticed that preparation of the catalyst in that case involving preparation of nanodendrites is less straight forward.

**Figure 5:**
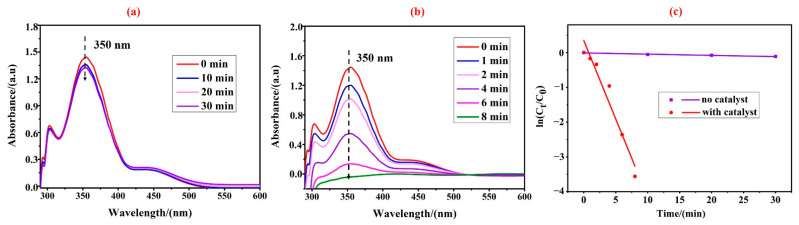
UV-vis spectra for catalytic reduction of Cr(VI) aqueous solution. (**a**) Treated with only formic acid (1.0 mL). (**b**) Treated with 1.0 cm Pd@wood catalyst, (**c**) the corresponding plots of ln(C_t_/C_0_) versus reaction time.

The reduction of Cr(VI) was carried out in the presence of formic acid at 50 °C to further investigate the catalytic performance of the prepared Pd@wood catalyst, as shown in Figure 5. The peak at 350 nm was assigned as the absorption peak of Cr(VI) [44]. There was no significant change in the concentration of Cr(VI) after 30 min with formic acid only (Figure 5a). This suggested that Cr(VI) was not reduced in the absence of the catalyst. After adding the Pd@wood catalyst, the intensity of the absorption peak gradually decreased and disappeared completely after 8 min. This showed that Cr(VI) was totally reduced (Figure 5b). For the reduction of Cr(VI), the rate constant k (s^−1^) is calculated based on the equation: ln(C_t_/C_0_) = kt, where t (min) is the reaction time, and C_t_ and C_0_ represent the concentration of Cr(VI) at the reaction time t and the initial time t_0_, respectively [45]. It was observed that the reaction rate constant was 7.53 × 10^−3^ s^−1^ for the wood composite catalyst with a length of 1.0 cm and Pd loading of 0.164 mg from the kinetic curve of Cr(VI) reduction (Figure 5c). In order to compare with the reported catalyst, the turnover frequency (TOF) is calculated based on the equation: TOF = (n(Cr(VI)))/(t × n(Pd)), where n(Cr(VI)) is the number of moles of Cr(VI) converted (mol), t represents the reaction time (min) and n(Pd) refers to the number of moles of Pd active sites (mol) [46]. The calculated turnover frequency of Pd was 2.03 min^−1^. Comparison of different catalyst systems on the reduction of Cr(VI) is shown in Table 2. It was observed that a shorter time for complete reduction of Cr(VI) was required and a higher TOF values was exhibited in our catalyst with Pd loading of 6.16 mol%, compared to other catalytic systems. It indicated that the prepared Pd@wood catalyst exhibited more excellent catalytic activity for the reduction of Cr(VI).

Furthermore, after the reaction, the wood block was taken out and ultrasonically cleaned at least three times alternately with acetone and deionized water, and then dried in an oven at 40 °C. The reusability of the catalyst for the Cr(VI) reduction reaction was then further investigated. In the subsequent four cycle reactions, the reduction efficiency of the catalyst for Cr(VI) could reach 99%, 98%, 96% and 93%, respectively, and the time required for the reaction increased from 8 min to 15 min. It indicated that the as-prepared catalyst had good reusability due to the internal structure and channels of the wood block providing good attachment points for Pd. A hot filtration test for the Pd@wood catalyst was performed as follows. A fresh 1.0 cm wood block catalyst was added to deionized water and magnetically stirred at 50 °C for 1 h at 800 rpm. Then the above solution was taken and added to an aqueous solution of Cr(VI) for reaction under the same conditions. The results showed that no Cr(VI) was reduced, which indicates that no Pd nanoparticles were leached during the reaction under mild conditions, confirming the good stability and reusability of the catalyst.

## 4. Conclusions

The Pd@wood catalyst with excellent catalytic performance and reproducible usability was successfully prepared. Meanwhile, microwave-assisted thermal synthesis of Pd nanoparticles was used as a model protocol for an efficient method for wood functionalization. The results showed that deep integration of Pd nanoparticles into the wood internal structure could be achieved, which has been confirmed by SEM and EDS images. The high flexibility and wide applicability of microwave-assisted synthesis in preparing materials with different dimensions provide a highly versatile and energy-efficient strategy for future integration of different functions into wood cell wall structures. Furthermore, for two model reactions of the reduction of 4-nitrophenol and Cr(VI), the Pd@wood catalyst shows 95.4 g^−1^ s^−1^ M^−1^ of normalized rate constant k_norm_ and 2.03 min^−1^ of the TOF, respectively. These two values are higher than most other reports, which shows that the prepared Pd@wood has excellent catalytic activity. It not only increases the high-value applications of wood through functionalization, but also has important implications for industrial wastewater treatment.

## Figures and Tables

**Table 1:** Comparison of different catalyst systems on the reduction of 4-nitrophenol.

Catalyst	Catalyst Mass (mg)	NaBH_4_ Concentration (mM)	k_app_ (s^−1^)	k_norm_ (g^−1^ s^−1^ M^−1^)	Ref.
Pd@wood	Pd (0.164)	282	4.41 × 10^−3^	95.4	This work
Ag@lignin	Ag (16.2)	500	2.30 × 10^−2^	2.8	[40]
PdND1	Pd (0.04)	77	3.95 × 10^−3^	1274.4	[41]
Cu NPs	Cu (12.5)	9.9	1.58 × 10^−3^	12.7	[38]
Ag-lignin/LCG1	---	10.0	2.19 × 10^−3^	---	[39]
Pt black	Pt (0.05)	203	0.70 × 10^−3^	69.0	[43]
Au@citrate	Au (0.05)	165	0.30 × 10^−3^	27.6	[43]
Ag dendrites	Ag (1.0)	36.4	2.51 × 10^−3^	68.9	[42]

Reaction conditions: 4-nitrophenol (0.807 mM, 5 mL), NaBH_4_ (0.282 M), Pd (0.164 mg, 1.54 × 10^−3^ mmol), 25 °C, using the wood catalyst with a length of 1.0 cm.

**Table 2:** Comparison of different catalyst systems on the reduction of Cr(VI).

Catalyst	Temperature (°C)	Time (min)	Rate Constant (k/min^−1^)	Rate Constant (k/s^−1^)	TOF (min^−1^)	Ref.
Pd@wood	50	8	0.452	7.53 × 10^−3^	2.03	This work
Pd-γ-Al_2_O_3_	50	40	0.085	1.42 × 10^−3^	1.03	[29]
Pd@Pro-ESM	45	26	0.133	2.22 × 10^−3^	2.6 × 10^−4^	[47]
Co-RGO_10_	25	9	0.474	7.90 × 10^−3^	4.9 × 10^−2^	[48]
Ni@GE-Cu_0.75_	25	15	0.344	5.73 × 10^−3^	2.4 × 10^−2^	[49]
Ni-RGO_10_	25	4	0.309	5.15 × 10^−3^	1.4 × 10^−2^	[50]

Reaction conditions: K_2_Cr_2_O_7_ (5 mmol/L, 5 mL), formic acid (1 mL), Pd (0.164 mg, 6.16 mol%).

## Data Availability

The raw data are not publicly available at this time but may be obtained from the authors upon reasonable request.

## Supplementary Materials

The following supporting information can be downloaded at: https://www.mdpi.com/article/10.3390/nano13172491/s1, Figure S1. The power, temperature and pressure of the whole process in the Microwave Synthesis Reactor to synthesize the Pd@wood catalyst. Figure S2. The size distribution of Pd nanoparticles. Table S1. The size distribution, mean size, amount and the frequencies of Pd nanoparticles in Figure S2a. Table S2. The size distribution, mean size, amount and the frequencies of Pd nanoparticles in Figure S2b. Figure S3. SEM images of the internal structure of Pd@wood catalyst. Figure S4. (a): Catalytic reduction of 4-nitrophenol by treated with wood catalysts of three different lengths (0.5, 1.0, 1.5 cm), and adsorption of 4-nitrophenol by 1.0 cm wood without Pd. (b): Kinetic curves corresponding plots of ln(C_t_/C_0_) versus reaction time. Figure S5. ^1^H and ^13^C NMR spectrum of 4-aminophenol.
