# Supplementary material for: Microwave-Assisted Synthesis of Pd Nanoparticles into Wood Block (Pd@wood) as Efficient Catalyst for 4-Nitrophenol and Cr(VI) Reduction
Source: Nanomaterials (Basel). 2023 Sep 4;13(17):2491. doi: 10.3390/nano13172491 (PMC10490320; doi:10.3390/nano13172491)
Supplement: Supplementary file 1 [file nanomaterials-13-02491-s001.zip › nanomaterials-2551872-supplementary.pdf]

Supporting Information

Microwave-assisted Synthesis of Pd Nanoparticles into Wood Block (Pd@wood) as Efficient Catalyst for 4-nitrophenol and Cr(VI) Reduction

Zhao Zhang[1], Arnaud Besserer[2], Christophe Rose[3], Nicolas Brosse[2], Vincent Terrasson[1,*], Erwann Guénin[1,*]

[1] Université de Technologie de Compiègne, ESCOM, TIMR (Integrated Transformations of Renewable Matter), Centre de Recherche Royallieu-CS 60 319-60 203 Compiègne Cedex, France.

[2] Université de Lorraine, INRAE, LERMAB, F-54000 Nancy, France.

[3] Centre INRAE-Grand Est-Nancy, UMR SYLVA-SILVATECH pole IM3, 54280 Champenoux, France.

[*] Correspondence: Erwann Guénin, E-mail: erwann.guenin@utc.fr; Tel.: +33-344-234-584; Vincent Terrasson, E-mail: v.terrasson@escom.fr

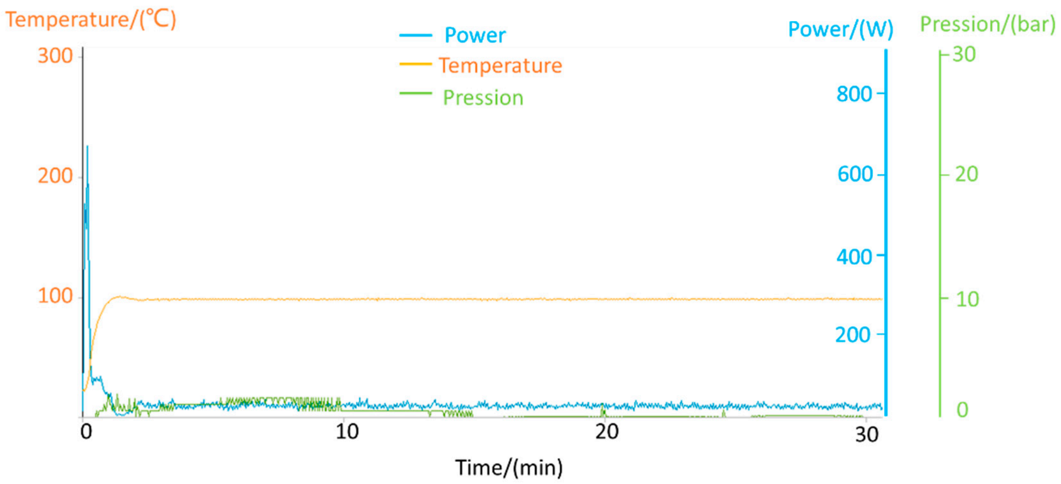

Figure S1. The power, temperature and pressure of the whole process in the Microwave Synthesis Reactor to synthesize the Pd@wood catalyst.

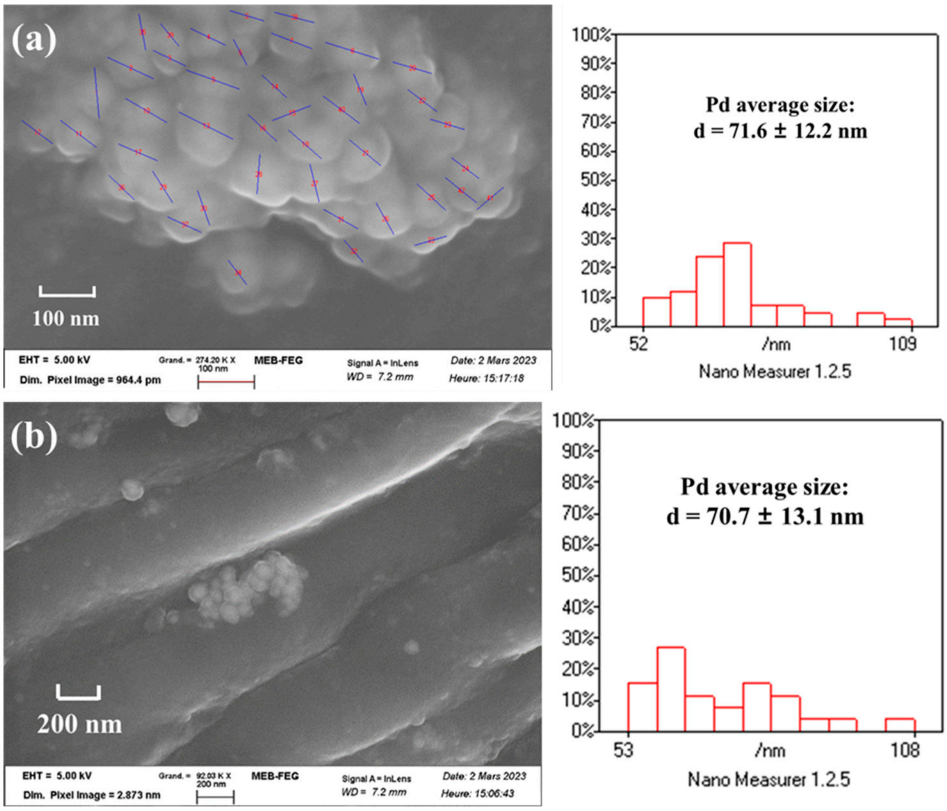

Figure S2. The size distribution of Pd nanoparticles.

Table S1. The size distribution, mean size, amount and the frequencies of Pd nanoparticles in Figure S2(a).

Distr./nm	Mean/nm	Amount	Freq.
52-57.7	54.85	4	9.52%
57.7-63.4	60.55	5	11.90%
63.4-69.1	66.25	10	23.81%
69.1-74.8	71.95	12	28.57%
74.8-80.5	77.65	3	7.14%
80.5-86.2	83.35	3	7.14%
86.2-91.8	89.05	2	4.76%
91.8-97.6	94.75	0	0.00%
97.6-103.3	100.45	2	4.76%
103.3-109	106.15	1	2.38%

Table S2. The size distribution, mean size, amount and the frequencies of Pd nanoparticles in Figure S2(b).

Distr./nm	Mean/nm	Amount	Freq.
53-58.5	55.75	4	15.38%
58.5-64	61.25	7	26.92%
64-69.5	66.75	3	11.54%
69.5-75	72.25	2	7.69%
75-80.5	77.75	4	15.38%
80.5-86	83.25	3	11.54%
86-91.5	88.75	1	3.85%
91.5-97	94.25	1	3.85%
97-102.5	99.75	0	0.00%
102.5-108	105.25	1	3.85%

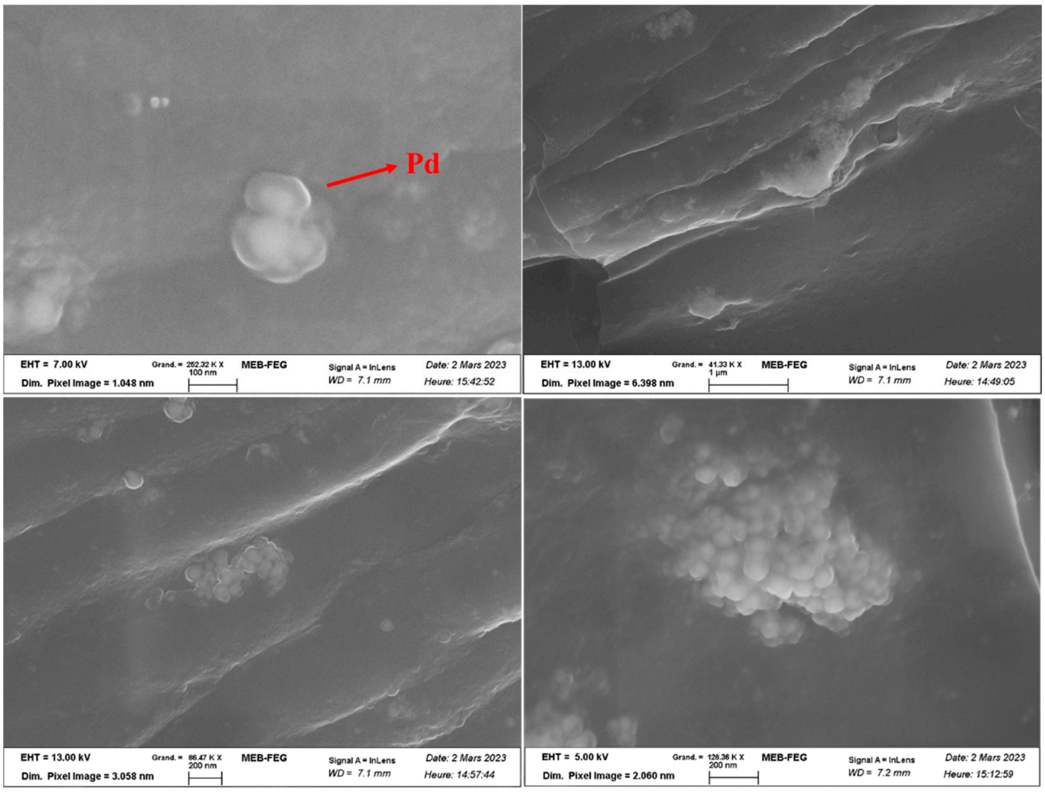

Figure S3. SEM images of the internal structure of Pd@wood catalyst.

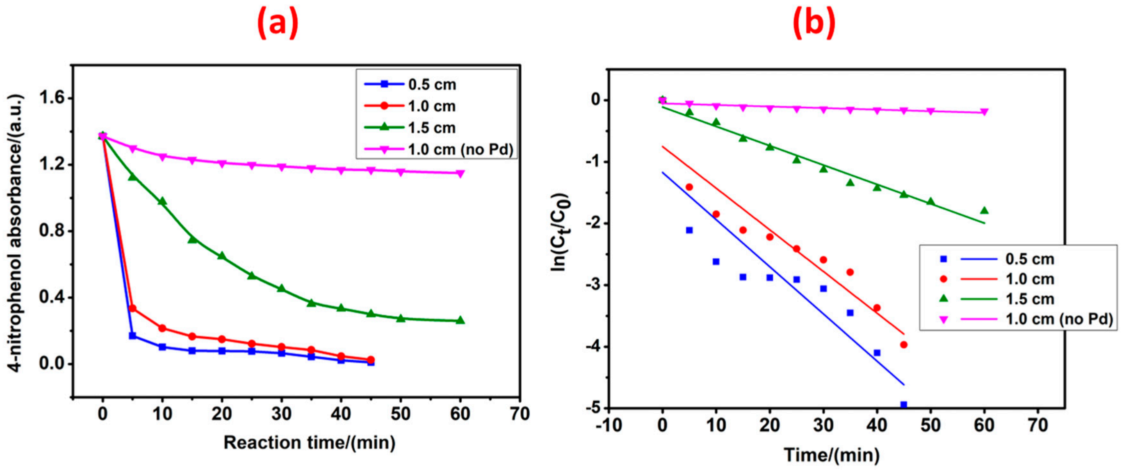

Figure S4. (a): Catalytic reduction of 4-nitrophenol by treated with wood catalysts of three different lengths (0.5, 1.0, 1.5 cm), and adsorption of 4-nitrophenol by 1.0 cm wood without Pd. (b): Kinetic curves corresponding plots of $\ln(C_t/C_0)$ versus reaction time.

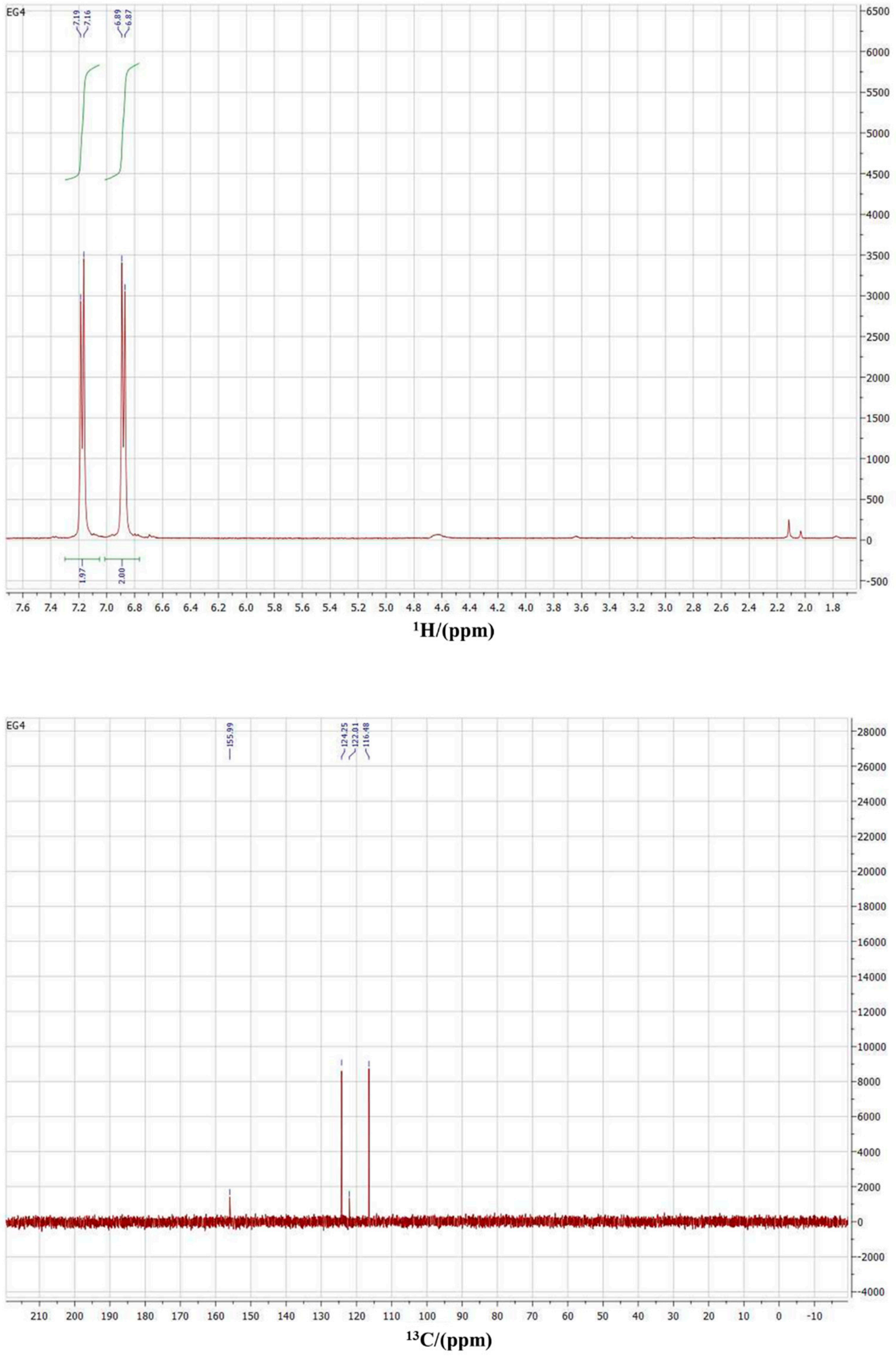

Figure S5. ^{1}H and ^{13}C NMR spectrum of 4-aminophenol.
